# Oncotype DX in Breast Cancer Management: Insights and Outcomes From the United Arab Emirates

**DOI:** 10.7759/cureus.56535

**Published:** 2024-03-20

**Authors:** Mouza A Ameri, Nandan M Shanbhag, Abdulrahman Bin Sumaida, Jawaher Ansari, Diaeddine A Trad, Emad A Dawoud, Khalid Balaraj

**Affiliations:** 1 Surgery, Tawam Hospital, Al Ain, ARE; 2 College of Medicine and Health Sciences, United Arab Emirates University, Al Ain, ARE; 3 Oncology/Radiation Oncology, Tawam Hospital, Al Ain, ARE; 4 Oncology/Palliative Care, Tawam Hospital, Al Ain, ARE; 5 Oncology, Tawam Hospital, Al Ain, ARE

**Keywords:** survival analysis, retrospective cohort study, recurrence risk personalized treatment, genomic testing, predictive accuracy, uae, oncotype dx, breast cancer

## Abstract

Introduction

Breast cancer remains the most significant cancer affecting women worldwide, with an increasing incidence, especially in developing regions. The introduction of genomic tests like Oncotype DX has revolutionized personalized treatment, allowing for more tailored approaches to therapy. This study focuses on the United Arab Emirates (UAE), where breast cancer is the leading cause of cancer-related deaths among women, aiming to assess the predictive accuracy of the Oncotype DX test in categorizing patients based on recurrence risk.

Materials and methods

A retrospective cohort study was conducted on 95 breast cancer patients diagnosed at Tawam Hospital between 2013 and 2017 who underwent Oncotype DX testing. Data on patient demographics, tumor characteristics, treatment details, and Oncotype DX scores were collected. Survival analysis was performed using the Kaplan-Meier method, with the chi-square goodness of fit test assessing the model’s adequacy.

Results

The cohort’s age range was 27-71 years, with a mean age of 50, indicating a significant concentration of cases in the early post-menopausal period. The Oncotype DX analysis classified 55 patients (57.9%) as low risk, 29 (30.5%) as medium risk, and 11 (11.6%) as high risk of recurrence. The majority, 73 patients (76.8%), did not receive chemotherapy, highlighting the test’s impact on treatment decisions. The survival analysis revealed no statistically significant difference in recurrence rates across the Oncotype DX risk categories (p = 0.268231).

Conclusion

The Oncotype DX test provides a valuable genomic approach to categorizing breast cancer patients by recurrence risk in the UAE. While the test influences treatment decisions, particularly the use of chemotherapy, this study did not find a significant correlation between Oncotype DX risk categories and actual recurrence events. These findings underscore the need for further research to optimize the use of genomic testing in the UAE’s diverse patient population and enhance personalized treatment strategies in breast cancer management.

## Introduction

Breast cancer remains the most prevalent cancer affecting women globally, with over two million new cases diagnosed in 2020 alone [1]. It accounts for almost a quarter of all cancer cases among women, and 685,000 people died due to breast cancer in 2018, highlighting a significant public health challenge across diverse healthcare systems [2]. Despite advancements in diagnosis and treatment contributing to decreased mortality rates in developed countries, the incidence continues to rise, particularly in developing regions [3]. This disparity highlights the urgent need for enhanced screening, early detection, and accessible treatment options worldwide to mitigate the global burden of breast cancer [4].

In the United Arab Emirates (UAE), breast cancer is the leading cause of cancer-related deaths among women, mirroring global trends [5]. The country has witnessed a rising incidence of breast cancer, prompting national efforts to improve awareness, screening, and diagnostics [6]. The UAE’s healthcare authorities have launched several initiatives, including nationwide screening campaigns and the establishment of specialized treatment centers, to reduce mortality through early detection [7]. Despite these efforts, challenges remain in addressing late-stage diagnosis rates, influenced by cultural stigmas and health literacy levels [8]. The UAE’s demographic composition is unique, with expatriates constituting approximately 80% of the population. This diverse expat community comes from over 200 countries, bringing varied cultural, dietary, and genetic backgrounds that influence health outcomes and disease prevalence, including breast cancer [9]. Healthcare strategies must, therefore, be tailored to address this heterogeneity, ensuring inclusive public health messaging and access to preventive services [10]. Moreover, the transient nature of the expat population poses additional challenges in implementing long-term healthcare interventions and tracking cancer epidemiology trends among this group.

Prognostic factors in breast cancer are indicators of the disease’s likely course and patient survival prospects [11]. These include tumor size, lymph node involvement, histological grade, and molecular subtype [12]. For instance, smaller tumors with no lymph node involvement generally have a better prognosis [13]. Similarly, breast cancers that are low-grade and express hormone receptors tend to have a more favorable outcome [14]. Understanding these factors is essential for staging the disease, guiding treatment choices, and providing patients with informed prognostic information. Predictive factors in breast cancer are crucial in guiding treatment decisions and predicting response to therapy [15]. Key predictive factors include hormone receptor status (estrogen and progesterone receptors, PRs), human epidermal growth factor receptor 2 (HER2) status, and gene expression profiles [16]. Estrogen and PRs often indicate a favorable response to hormone therapy, while HER2-positive cancers may respond well to targeted HER2 therapies [17]. Additionally, genomic tests like Oncotype DX can predict chemotherapy benefits, enabling personalized treatment plans that optimize outcomes and minimize unnecessary side effects [18]. Oncotype DX is a genomic test that analyzes the expression of a group of cancer-related genes in a breast tumor, providing a Recurrence Score (RS) that predicts the likelihood of cancer recurrence [19]. This score helps decide the benefit of chemotherapy for hormone receptor-positive, HER2-negative breast cancer patients [20]. Oncotype DX has revolutionized personalized breast cancer treatment, enabling clinicians to tailor therapies based on individual genetic profiles, thereby optimizing outcomes and avoiding overtreatment [21].

Endocrine therapy is a fundamental component in the management of hormone receptor-positive breast cancer, with the primary objective of inhibiting the proliferative influence of estrogen and progesterone on the cancer cells [22]. Widely utilized pharmaceutical agents such as tamoxifen and aromatase inhibitors have effectively diminished recurrence risk and enhanced survival outcomes [23]. The selection and duration of this therapeutic approach are contingent upon the unique characteristics of the cancer and the menopausal status of the patient, emphasizing the necessity for individualized treatment plans in the comprehensive management of breast cancer [24]. Chemotherapy is a pivotal intervention in the therapeutic approach to breast cancer, especially in cases characterized by aggressive behavior or advanced disease progression. Its mechanism of action involves targeting rapidly proliferating cancer cells, albeit with the potential to impact healthy cells, thereby resulting in adverse effects [25]. The decision to administer chemotherapy, as well as the selection of specific agents, is informed by various factors, encompassing the stage of the cancer, its subtype, and the presence of particular genetic markers [26]. The evolution of chemotherapy protocols has led to substantial enhancements in patient outcomes, thereby solidifying its status as an indispensable component of the armamentarium for breast cancer treatment [27]. Radiotherapy is a common treatment modality for breast cancer, utilized mainly post-surgery as either a part of the breast conservation strategy or as post-mastectomy radiotherapy [28]. It significantly reduces the risk of local recurrence and improves survival rates [29]. Precision in targeting the tumor site while sparing surrounding healthy tissue is a focus of ongoing technological advancements in radiotherapy, aiming to minimize side effects and enhance treatment efficacy [30].

Assessing the risk of breast cancer recurrence is crucial for tailoring follow-up care and determining the need for adjuvant therapies. Factors considered in recurrence risk assessment include tumor size, grade, lymph node status, and molecular characteristics. Tools like the Oncotype DX test offer a genomic approach to evaluate recurrence risk, providing valuable information to guide treatment decisions and follow-up strategies.

## Materials and methods

Study design and population

This study employed a retrospective cohort design to evaluate the predictive accuracy of the Oncotype DX test in categorizing breast cancer patients into distinct risk groups based on recurrence likelihood. The cohort comprised patients diagnosed with breast cancer at Tawam Hospital between 2013 and 2017. Eligibility criteria included patients with histologically confirmed invasive breast cancer who underwent Oncotype DX testing as part of their clinical management. Patients were excluded if they had metastatic disease at diagnosis, had incomplete medical records, or had not received Oncotype DX testing.

Oncotype DX risk categorization

The Oncotype DX test, a genomic assay, quantifies the expression of 21 genes in tumor tissue, producing a RS that stratifies patients into low (<18), intermediate (18-31), and high (>31) risk of recurrence categories. This RS guides adjuvant therapy decisions, aiming to personalize treatment approaches based on genetic risk profiles.

Data collection

Patient demographics, tumor characteristics (size, grade, and hormone receptor status), treatment details (surgery type, chemotherapy, radiotherapy, and endocrine therapy), and Oncotype DX scores were extracted from electronic health records. Survival data, including time to recurrence, was also collected, with follow-up data censored at the last known date of contact or date of recurrence, whichever came first.

Statistical analysis

The primary outcome was the validation of the Oncotype DX risk categories against actual recurrence events within the cohort. Survival analysis was conducted using the Kaplan-Meier method, with log-rank tests comparing survival curves across risk groups. The chi-square goodness of fit test assessed the model’s adequacy, with a p-value <0.05 considered statistically significant. The effect size was calculated to quantify the magnitude of differences between observed and expected outcomes, providing insight into their practical significance. Statistical analyses were performed using R-Studio and online statistical software.

Ethical considerations

The Tawam Human Research Ethics Committee reviewed and approved the study protocol, ensuring compliance with ethical standards for research involving human subjects. Patient confidentiality was maintained according to regulations, with all data anonymized before analysis.

## Results

**Figure 1:**
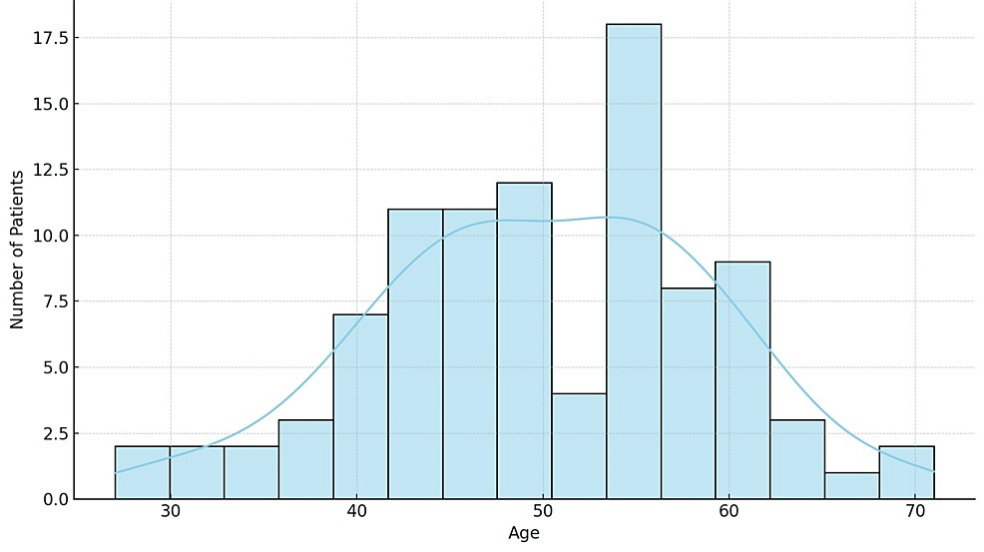
Age distribution x-axis: age in years, y-axis: number of patients N = 95

The study encompasses a total of 95 patients diagnosed with breast cancer. The ages at diagnosis ranged from 27 to 71 years, with a mean age at diagnosis of approximately 50 years, indicating that the midpoint of age distribution lies in the early post-menopausal period, which is a critical time for breast cancer screening and diagnosis. The standard deviation of the ages was approximately nine years, reflecting a moderate spread around the mean age. This variability suggests that while there is a concentration of cases around the mean, many patients are diagnosed younger and older than this central value (Figure 1).

Consequently, 49 patients were Emiratis, constituting 51.6% of the study population. In contrast, 46 patients were identified as non-nationals, accounting for 48.4% of the dataset. The analysis of menopausal status among the breast cancer patients in the study revealed a diverse distribution across different menopausal stages, with equidistribution among pre- and post-menopausal status at 42 patients (44.2%) and 43 patients (45.3%), respectively. Only 10 patients (10.5%) were perimenopausal, referring to those in the transitional phase leading up to menopause.

**Figure 2:**
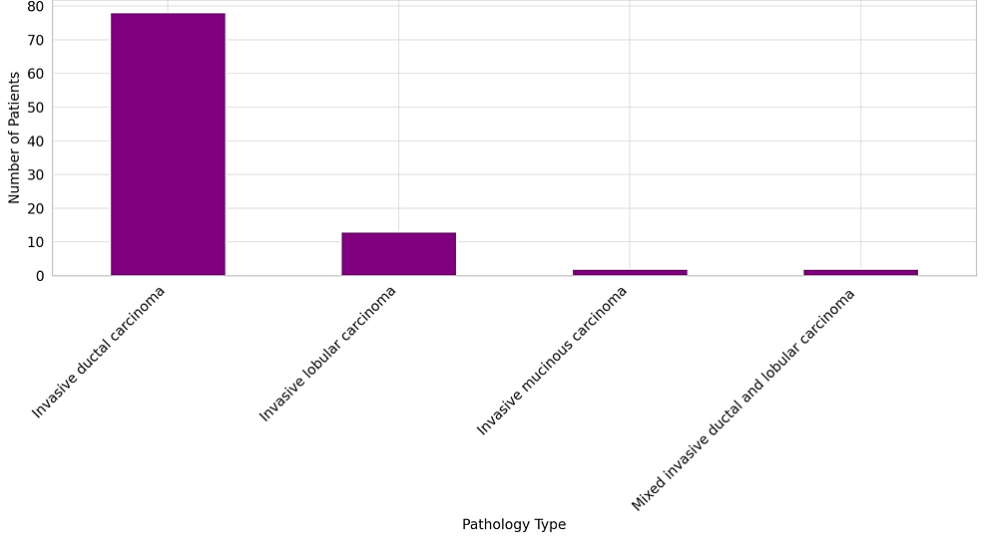
Pathology of breast cancer N = 95

The study meticulously categorized the pathology of breast cancer, highlighting the histopathological diversity inherent in breast cancer diagnoses. The analysis delineated four primary pathology types: invasive ductal carcinoma (IDC), invasive lobular carcinoma (ILC), invasive mucinous carcinoma (IMC), and mixed invasive ductal and lobular carcinoma (mixed). IDC emerged as the predominant pathology, diagnosed in 78 patients, which accounts for 82.1% of the cases. ILC, identified in 13 patients, represents 13.7% of the study population. IMC was observed in two patients, making up 2.1% of the cases. Similarly, mixed variety, featuring characteristics of both IDC and ILC, was also found in two patients, constituting another 2.1% of the cohort (Figure 2).

**Figure 3:**
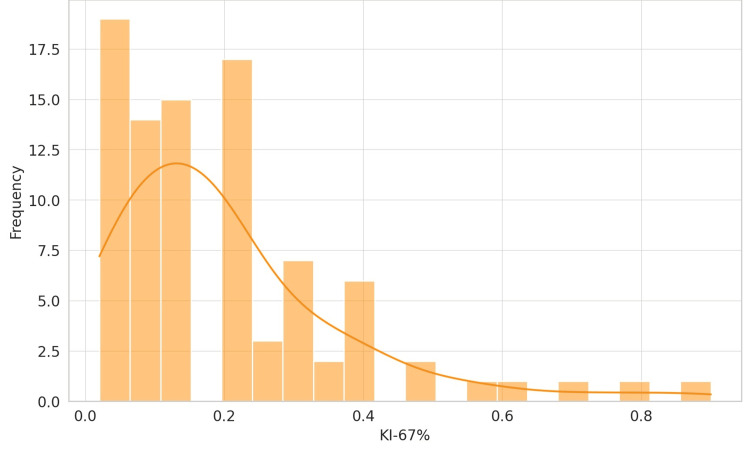
Distribution of the KI-67 proliferation index in breast cancer patients The KI-67 is depicted in percentage; hence, the values on the x-axis are percentage 0.2 to be read as 20%, 0.4 as 40%, and so forth. N = 95

An in situ component was observed in two patients (2.1%), while the majority, 93 patients (97.9%), did not exhibit this feature. Estrogen receptor (ER) positivity was identified in 93 patients (97.9%), with only two patients (2.1%) being ER-negative. PR positivity was found in 86 patients (90.5%), whereas nine patients (9.5%) were PR-negative. All 95 patients (100%) in the study were HER2-negative. The evaluation of tumor grade within the patient cohort reveals a distribution favoring Grade 2 tumors, with the following counts and corresponding percentages: Grade 1 (Well-differentiated): 16 patients (16.8%); Grade 2 (Moderately differentiated): 67 patients (70.5%); and Grade 3 (Poorly differentiated): 12 patients (12.6%). The KI-67 proliferation index variable is a marker for cellular proliferation. The analysis reveals a diverse range of values, indicative of varying tumor aggressiveness within the cohort. Nineteen different KI-67% values were reported, with a KI-67 of 20% observed in 17 patients (17.89%) (Figure 3).

Forty-six patients (48.4%) did not undergo genetic testing, and of the 49 patients who underwent the testing, 38 (40%) tested negative, three (3.2%) had BRCA2 mutations, two (2.1%) had mutations in both BRCA1 and BRCA2, and one (1.1%) had a BRCA1 mutation. Additionally, for other genetic mutations, nine (9.5%) were identified with mutations in genes such as NF1, PIK3CA, and POLE.

In this cohort, surgical treatment strategies for breast cancer were closely analyzed, revealing significant insights into the clinical approaches adopted. The distribution of cancer laterality showed a nearly even split between the left (46.3%, 44 patients) and right (45.3%, 43 patients) breasts, with a smaller proportion of cases (8.4%, eight patients) being bilateral, affecting both breasts. When it came to local surgical interventions, a majority of the patients (68.4%, 65 patients) underwent breast-conserving lumpectomy; conversely, mastectomy was performed in 31.6% (30 patients) of the cohort, reflecting a significant yet lesser preference compared to lumpectomy. Regarding nodal surgery, the sentinel lymph node biopsy was the predominant choice, performed in 83.2% (78 patients) of cases to evaluate the initial lymph nodes for cancer spread. In contrast, axillary lymph node dissection, a procedure to remove multiple lymph nodes from the axillary area, was conducted in 16.8% (16 patients) of the cases. A significant majority, 79 patients (83.2%), had no lymphadenopathy, suggesting the absence of clinically detectable abnormal lymph nodes at diagnosis, with 16 patients (16.8%) having a lymph node-positive disease. Among patients with node involvement, the average number of positive nodes was approximately 1.88, with a standard deviation of 1.36. The minimum number of positive nodes recorded was one, and the maximum was five. For the same subset, the average number of nodes removed during surgical intervention was 9.5, with a standard deviation of 6.87.

**Figure 4:**
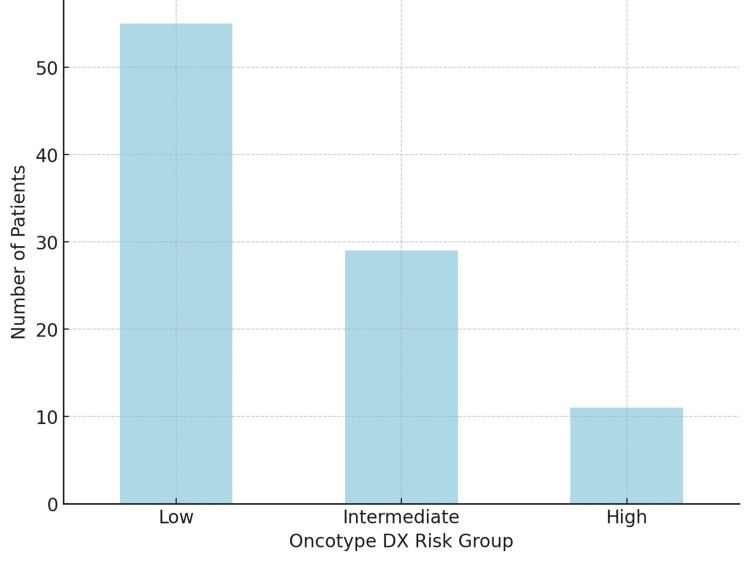
Oncotype DX risk groups Oncotype DX score was categorized into three risk groups to assess the risk of recurrence in breast cancer patients: low (0 to <18), intermediate (18 to 31), and high (>31). N = 95

The Oncotype DX analysis yielded the following distribution: 55 patients, constituting 57.9% of the cohort as low risk; 29 patients (30.5%) as medium risk; and 11 patients (11.6%) as high risk (Figure 4).

The majority of the patients, 73 (75.8%), did not receive chemotherapy, and 22 (23.2%) received chemotherapy. According to the Oncotype DX risk stratification, two (2.1%) were low risk, and 10 (10.52%) were intermediate and high risk each among those who received chemotherapy. The distribution of radiotherapy doses and the number of fractions reflect standard treatment regimens, with 75 patients (78.9%) receiving radiotherapy with 40 Gy or 50 Gy, typically administered in 15 or 25 fractions (sessions). Of these, 46 patients (48.4%) received radiotherapy with an additional boost, with 34 patients (35.8%) and 12 patients (12.6%) receiving 10 Gy and 16 Gy as boosts, respectively.

**Figure 5:**
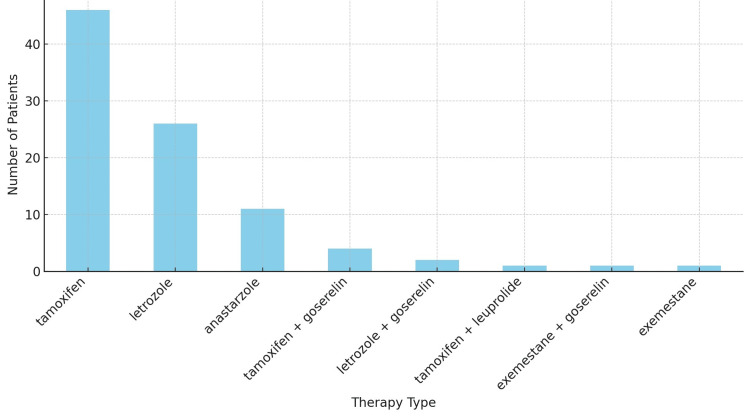
Initial endocrine therapy Three patients did not receive endocrine therapy. N = 95

Only three patients (3.8%) did not receive endocrine therapy. Of the 92 patients (96.84%) who received adjuvant endocrine therapy, 46 (48.4%) initiated treatment with tamoxifen, 26 patients (27.4%) with letrozole, 11 patients (11.6%) with anastrozole, and one patient (1.1%) with exemestane, with the rest starting a combination regimen including tamoxifen + goserelin in four patients (4.2%), letrozole + goserelin in two patients (2.1%), tamoxifen + leuprolide and exemestane + goserelin in one patient (1.1%) each (Figure 5).

**Figure 6:**
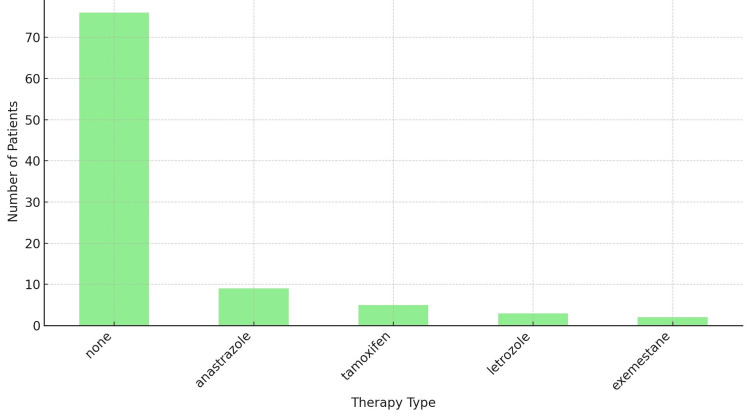
Endocrine switch None reflects that the majority; 76 patients (80%), did not switch their initial endocrine regimen. N = 95

The majority, 76 patients (80%), did not switch their adjuvant endocrine therapy. Nine patients (9.5%) changed to anastrozole, and five patients (5.3%) switched to tamoxifen. Fewer patients switched to letrozole (three patients; 3.2%) and exemestane (two patients; 2.1%) (Figure 6).

**Figure 7:**
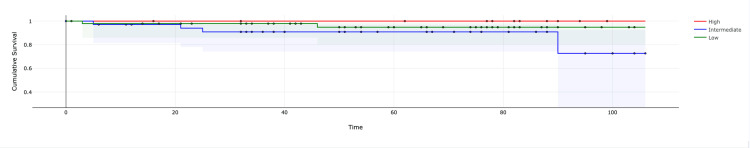
Kaplan-Meier recurrence-free survival curve with confidence interval for the three risk groups The Oncotype DX score was categorized into three risk groups to assess the risk of recurrence in breast cancer patients: low (0 to <18), intermediate (18 to 31), and high (>31). Solid-colored dots represent the censored data.

Kaplan-Meier recurrence-free survival analysis was conducted for the three Oncotype DX risk groups (Figure 7, Table 1, Table 2, Table 3).

**Table 1:** Survival table – high risk Event of interest (Dt): 0 - no recurrence, 1 – recurrence; Censored event (Ct): the event of interest did not happen since the subject left the experiment before the ending time or due to the termination of the study; nt: number of participants that did not have an event yet (event of interest or censored event); proportion surviving interval (Pt): the proportion of survival participants between period t-1 and period t; survival rate (St cumulative survival/survival function): the proportion of survival participants from period 0 to period t; Sum = ΣDt/nt(nt - Dt); S.Et: the standard deviation of the survival rate. S.E = St√(Sum); Lower: lower bound of St confidence interval; Upper: upper bound of St confidence interval N = 95

Time (months)	Events (Dt)	Censored (Ct)	nt	Survival rate (St)	Incidence rate (Ft)	Pt	Sum = ΣDt/nt(nt-Dt)	S.Et	Lower	Upper	Dt/nt(nt-Dt)
0	0	0	11	1	0	0	0	0	1	1	0
16	0	1	11	1	0	1	0	0	1	1	0
32	0	1	10	1	0	1	0	0	1	1	0
62	0	1	9	1	0	1	0	0	1	1	0
77	0	1	8	1	0	1	0	0	1	1	0
78	0	1	7	1	0	1	0	0	1	1	0
82	0	1	6	1	0	1	0	0	1	1	0
83	0	1	5	1	0	1	0	0	1	1	0
88	0	1	4	1	0	1	0	0	1	1	0
90	0	1	3	1	0	1	0	0	1	1	0
94	0	1	2	1	0	1	0	0	1	1	0
99	0	1	1	1	0	1	0	0	1	1	0

**Table 2:** Survival table – intermediate risk Event of interest (Dt): 0 - no recurrence, 1 - recurrence; Censored event (Ct): the event of interest did not happen since the subject left the experiment before the ending time or due to the termination of the study; nt: number of participants that did not have an event yet (event of interest or censored event); proportion surviving interval (Pt): the proportion of survival participants between period t-1 and period t; survival rate (St cumulative survival/survival function): the proportion of survival participants from period 0 to period t; Sum = ΣDt/nt(nt - Dt); S.Et: the standard deviation of the survival rate. S.E = St√(Sum); Lower: lower bound of St confidence interval; Upper: upper bound of St confidence interval N = 95

Time (months)	Events (Dt)	Censored (Ct)	nt	Survival rate (St)	Incidence rate (Ft)	Pt	Sum = ΣDt/nt(nt-Dt)	S.Et	Lower	Upper	Dt/nt(nt-Dt)
0	0	0	36	1	0	0	0	0	1	1	0
0	0	1	36	1	0	1	0	0	1	1	0
5	1	0	35	0.9714	0.0286	0.9714	0.0008	0.0282	0.814	0.9959	0.0008
6	0	1	34	0.9714	0.0286	1	0.0008	0.0282	0.814	0.9959	0
11	0	1	33	0.9714	0.0286	1	0.0008	0.0282	0.814	0.9959	0
12	0	1	32	0.9714	0.0286	1	0.0008	0.0282	0.814	0.9959	0
21	1	0	31	0.9401	0.0599	0.9677	0.0019	0.0412	0.7806	0.9847	0.0011
25	1	0	30	0.9088	0.0912	0.9667	0.0031	0.0503	0.7427	0.9697	0.0011
32	0	2	29	0.9088	0.0912	1	0.0031	0.0503	0.7427	0.9697	0
33	0	1	27	0.9088	0.0912	1	0.0031	0.0503	0.7427	0.9697	0
34	0	2	26	0.9088	0.0912	1	0.0031	0.0503	0.7427	0.9697	0
36	0	1	24	0.9088	0.0912	1	0.0031	0.0503	0.7427	0.9697	0
38	0	2	23	0.9088	0.0912	1	0.0031	0.0503	0.7427	0.9697	0
40	0	1	21	0.9088	0.0912	1	0.0031	0.0503	0.7427	0.9697	0
50	0	1	20	0.9088	0.0912	1	0.0031	0.0503	0.7427	0.9697	0
51	0	1	19	0.9088	0.0912	1	0.0031	0.0503	0.7427	0.9697	0
54	0	2	18	0.9088	0.0912	1	0.0031	0.0503	0.7427	0.9697	0
57	0	1	16	0.9088	0.0912	1	0.0031	0.0503	0.7427	0.9697	0
66	0	1	15	0.9088	0.0912	1	0.0031	0.0503	0.7427	0.9697	0
67	0	1	14	0.9088	0.0912	1	0.0031	0.0503	0.7427	0.9697	0
71	0	1	13	0.9088	0.0912	1	0.0031	0.0503	0.7427	0.9697	0
74	0	2	12	0.9088	0.0912	1	0.0031	0.0503	0.7427	0.9697	0
76	0	1	10	0.9088	0.0912	1	0.0031	0.0503	0.7427	0.9697	0
81	0	1	9	0.9088	0.0912	1	0.0031	0.0503	0.7427	0.9697	0
86	0	1	8	0.9088	0.0912	1	0.0031	0.0503	0.7427	0.9697	0
88	0	2	7	0.9088	0.0912	1	0.0031	0.0503	0.7427	0.9697	0
90	1	0	5	0.727	0.273	0.8	0.0531	0.1675	0.2688	0.9256	0.05
95	0	1	4	0.727	0.273	1	0.0531	0.1675	0.2688	0.9256	0
100	0	1	3	0.727	0.273	1	0.0531	0.1675	0.2688	0.9256	0
104	0	1	2	0.727	0.273	1	0.0531	0.1675	0.2688	0.9256	0
106	0	1	1	0.727	0.273	1	0.0531	0.1675	0.2688	0.9256	0

**Table 3:** Survival table – low risk Event of interest (Dt): 0 - no recurrence, 1 - recurrence; Censored event (Ct): the event of interest did not happen since the subject left the experiment before the ending time or due to the termination of the study; nt: number of participants that did not have an event yet (event of interest or censored event); proportion surviving interval (Pt): the proportion of survival participants between period t-1 and period t; survival rate (St cumulative survival/survival function): the proportion of survival participants from period 0 to period t; Sum = ΣDt/nt(nt - Dt); S.Et: the standard deviation of the survival rate. S.E = St√(Sum); Lower: lower bound of St confidence interval; Upper: upper bound of St confidence interval. N = 95

Time (months)	Events (Dt)	Censored (Ct)	nt	Survival rate (St)	Incidence rate (Ft)	Pt	Sum = ΣDt/nt(nt-Dt)	S.Et	Lower	Upper	Dt/nt(nt-Dt)
0	0	0	48	1	0	0	0	0	1	1	0
1	0	1	48	1	0	1	0	0	1	1	0
3	1	0	47	0.9787	0.0213	0.9787	0.0005	0.0211	0.8584	0.997	0.0005
14	0	1	46	0.9787	0.0213	1	0.0005	0.0211	0.8584	0.997	0
17	0	1	45	0.9787	0.0213	1	0.0005	0.0211	0.8584	0.997	0
21	0	1	44	0.9787	0.0213	1	0.0005	0.0211	0.8584	0.997	0
23	0	1	43	0.9787	0.0213	1	0.0005	0.0211	0.8584	0.997	0
32	0	1	42	0.9787	0.0213	1	0.0005	0.0211	0.8584	0.997	0
33	0	2	41	0.9787	0.0213	1	0.0005	0.0211	0.8584	0.997	0
34	0	1	39	0.9787	0.0213	1	0.0005	0.0211	0.8584	0.997	0
37	0	1	38	0.9787	0.0213	1	0.0005	0.0211	0.8584	0.997	0
38	0	1	37	0.9787	0.0213	1	0.0005	0.0211	0.8584	0.997	0
41	0	2	36	0.9787	0.0213	1	0.0005	0.0211	0.8584	0.997	0
42	0	1	34	0.9787	0.0213	1	0.0005	0.0211	0.8584	0.997	0
43	0	1	33	0.9787	0.0213	1	0.0005	0.0211	0.8584	0.997	0
46	1	0	32	0.9481	0.0519	0.9688	0.0015	0.0364	0.8038	0.9871	0.001
50	0	1	31	0.9481	0.0519	1	0.0015	0.0364	0.8038	0.9871	0
51	0	2	30	0.9481	0.0519	1	0.0015	0.0364	0.8038	0.9871	0
53	0	2	28	0.9481	0.0519	1	0.0015	0.0364	0.8038	0.9871	0
54	0	1	26	0.9481	0.0519	1	0.0015	0.0364	0.8038	0.9871	0
59	0	1	25	0.9481	0.0519	1	0.0015	0.0364	0.8038	0.9871	0
60	0	1	24	0.9481	0.0519	1	0.0015	0.0364	0.8038	0.9871	0
65	0	1	23	0.9481	0.0519	1	0.0015	0.0364	0.8038	0.9871	0
66	0	1	22	0.9481	0.0519	1	0.0015	0.0364	0.8038	0.9871	0
69	0	1	21	0.9481	0.0519	1	0.0015	0.0364	0.8038	0.9871	0
74	0	2	20	0.9481	0.0519	1	0.0015	0.0364	0.8038	0.9871	0
75	0	1	18	0.9481	0.0519	1	0.0015	0.0364	0.8038	0.9871	0
76	0	1	17	0.9481	0.0519	1	0.0015	0.0364	0.8038	0.9871	0
77	0	1	16	0.9481	0.0519	1	0.0015	0.0364	0.8038	0.9871	0
78	0	1	15	0.9481	0.0519	1	0.0015	0.0364	0.8038	0.9871	0
79	0	1	14	0.9481	0.0519	1	0.0015	0.0364	0.8038	0.9871	0
81	0	1	13	0.9481	0.0519	1	0.0015	0.0364	0.8038	0.9871	0
82	0	1	12	0.9481	0.0519	1	0.0015	0.0364	0.8038	0.9871	0
83	0	2	11	0.9481	0.0519	1	0.0015	0.0364	0.8038	0.9871	0
87	0	2	9	0.9481	0.0519	1	0.0015	0.0364	0.8038	0.9871	0
88	0	2	7	0.9481	0.0519	1	0.0015	0.0364	0.8038	0.9871	0
90	0	1	5	0.9481	0.0519	1	0.0015	0.0364	0.8038	0.9871	0
95	0	1	4	0.9481	0.0519	1	0.0015	0.0364	0.8038	0.9871	0
98	0	1	3	0.9481	0.0519	1	0.0015	0.0364	0.8038	0.9871	0
103	0	1	2	0.9481	0.0519	1	0.0015	0.0364	0.8038	0.9871	0
104	0	1	1	0.9481	0.0519	1	0.0015	0.0364	0.8038	0.9871	0

A log-rank test was calculated using the chi-squared goodness of fit test to check the null assumption model of equal survival distributions (Table 4, Table 5).

**Table 4:** Observed versus expected frequencies for the event Event: recurrence The Oncotype DX score was categorized into three risk groups to assess the risk of recurrence in breast cancer patients: low (0 to <18), intermediate (18-31), and high (>31.)

Group	Observed frequency	Expected frequency
High risk	0	0.85
Intermediate risk	4	2.19
Low risk	2	2.94

**Table 5:** Log-rank test summary table The log-rank test model assumes the events per subject are distributed evenly between the groups for log-rank test m = 0.

Chi-square variables	Values	Definition
k	3	Number of groups
N	95	Sample size
e	6	Event
χ²	2.6318	Chi-square test statistic
DF	2	Df = k - m -1 = 3 - 0 - 1 = 2
Phi effect (Φ)	0.6623	Φ = √(χ2/n)

The chi-square goodness of fit test yielded a p-value of 0.268231, substantially exceeding the conventional threshold for statistical significance set at p < 0.05. This outcome indicates that the probability of observing such data under the null hypothesis, which posits no difference between the model and observed groups, is 26.82%. The chi-square statistic of 2.631814 falls within the 95% acceptance region (critical value of 5.9915). An observed effect size of 0.66 highlights a considerable difference within the model.

## Discussion

The study encompasses a total of 95 patients diagnosed with breast cancer. The ages at diagnosis ranged from 27 to 71 years, with a mean age at diagnosis of approximately 50 years, indicating that the midpoint of age distribution lies in the early post-menopausal period, which is a critical time for breast cancer screening and diagnosis. The relationship between age at diagnosis, menopausal status, and breast cancer screening strategies is a critical area of research in oncology. Recent studies provide insights into how these factors impact breast cancer prognosis and the effectiveness of screening programs. Miglioretti et al. found that premenopausal women diagnosed with breast cancer following biennial vs annual screening mammography were more likely to have tumors with less favorable prognostic characteristics. This suggests that the frequency of screening and the timing of menopause can significantly impact breast cancer outcomes [31]. The American College of Radiology (ACR) emphasizes the importance of starting mammographic screening earlier for women at higher risk and highlights that supplemental screening modalities may benefit these women [32]. Studies indicate that menopausal status can significantly influence the risk and management of breast cancer. Postmenopausal women, especially those who are overweight or obese, have an increased risk of invasive breast cancer, highlighting the interplay between hormonal changes after menopause and cancer risk. Menopausal status is a critical factor in breast cancer risk, with different risk profiles for premenopausal and postmenopausal women. Studies have shown that the hormonal changes accompanying menopause can influence the development and progression of breast cancer. For instance, postmenopausal women often have breast cancers that are hormone receptor-positive, suggesting a link between hormone levels and cancer growth [33].

The analysis of menopausal status among the breast cancer patients in the study revealed a diverse distribution across different menopausal stages. The near-equal distribution of premenopausal and postmenopausal patients, along with a smaller segment of perimenopausal women, reflects the complex interplay between hormonal status and breast cancer. It emphasizes the necessity for tailored screening and treatment strategies that consider menopausal status and its associated physiological changes [34]. This distribution also highlights the importance of inclusive research and clinical practices that address the needs and risks associated with each menopausal stage, thereby optimizing care for all women, irrespective of their menopausal status [35].

The implementation of educational campaigns about menopause, menopausal hormone therapy, and breast cancer risks is essential for improving women’s knowledge and coping strategies for menopause symptoms and breast cancer risk. Such campaigns could significantly contribute to the early detection and management of breast cancer, particularly among postmenopausal women, who may be at increased risk [36]. The awareness and utilization of breast cancer screening programs among women in the UAE have been subjects of extensive study. Hegde et al. highlighted the need for improved awareness and practice of breast cancer screening methods among women in the region [37]. Research indicates cultural and sociodemographic factors significantly influence breast cancer knowledge, attitudes, and screening practices. For example, a study suggested that Emirati women, compared to non-nationals, may have different levels of awareness and attitudes towards breast cancer and its screening [38]. These differences highlight the need for culturally sensitive and tailored health education programs to address the unique needs of diverse populations within the UAE.

IDC emerged as the predominant pathology, diagnosed in 78 patients, which accounts for 82.1% of the cases. This finding is consistent with global statistics, where IDC is recognized as the most common form of breast cancer, characterized by cancer cells breaking through the ductal wall and invading the surrounding breast tissue [39]. ILC, identified in 13 patients, represents 13.7% of the study population. ILC originates in the lobules and is known for its distinctive pattern of spreading more diffusely through the breast tissue and potentially to distant sites.

The study meticulously evaluated crucial histopathological markers in breast cancer patients, including the presence of an in situ component and the status of ER, PR, and HER2. These markers are instrumental in guiding treatment decisions and prognostication [40]. The rarity of the in situ component in this cohort suggests that most cancers were invasive at the time of diagnosis, which has implications for treatment planning and prognosis [41]. ER positivity was identified in 93 patients (97.9%), with only two patients (2.1%) being ER-negative. The high prevalence of ER-positive cases signifies the potential effectiveness of hormone therapy in this cohort, given its pivotal role in managing ER-positive breast cancers [42]. PR positivity was found in 86 patients (90.5%), whereas nine patients (9.5%) were PR-negative. Like ER status, the predominance of PR-positive cases indicates the relevance of hormone receptor status in determining therapeutic strategies [43]. All patients in the study were HER2-negative. This uniformity in HER2 status highlights a specific patient population that does not benefit from HER2-targeted therapies but may be eligible for other treatment modalities [44]. These findings reflect the diagnostic and prognostic significance of histopathological markers in breast cancer [45]. The predominance of hormone receptor-positive and HER2-negative statuses in this cohort suggests that hormone therapy, rather than HER2-targeted therapies, was central to the management strategies for these patients. The minimal presence of an in situ component indicates a lower potential for localized treatment approaches, emphasizing the need for comprehensive management plans that address the invasive nature of the diagnosed cancers. Understanding the distribution of these markers is critical for clinicians in tailoring treatment plans that align with the molecular characteristics of cancer, thereby optimizing patient outcomes.

The study’s investigation into lymphadenopathy and lymph node involvement provides critical insights into the extent of disease among the patients. A significant majority, 79 patients (83.2%), were found to have no lymphadenopathy, suggesting the absence of clinically detectable abnormal lymph nodes at diagnosis. Conversely, 16 patients (16.8%) were diagnosed with lymphadenopathy, indicating the presence of abnormal lymph nodes that potentially reflect metastatic spread or localized disease involvement. Further analysis of lymph node involvement, specifically the number of positive nodes and the number of nodes removed, reveals that the average number of positive nodes among patients with node involvement was approximately 1.88, with a standard deviation of 1.36. The minimum number of positive nodes recorded was 1, and the maximum was 5, indicating variable disease spread within the lymphatic system. These statistics are based on the subset of patients for whom this data was available, highlighting the variability in disease extent among those with lymph node involvement. For the same subset, the average number of nodes removed during surgical intervention was 9.5, with a standard deviation of 6.87. This reflects a range from three to 25 nodes removed, illustrating the surgical approach to removing potentially affected lymph nodes for disease control and staging. The findings reflect the importance of lymph node evaluation in breast cancer diagnosis and treatment planning. The absence of lymphadenopathy in most patients suggests early detection or localized disease in a significant portion of the cohort. However, the presence of lymphadenopathy in a notable minority highlights the importance of comprehensive disease staging and the potential need for more aggressive treatment strategies, including surgery and adjuvant therapies. Moreover, the extent of lymph node removal, which varies widely among patients, reflects tailored surgical approaches based on individual disease characteristics and the principle of adequate disease control while minimizing morbidity [46]. These insights into lymphadenopathy and lymph node involvement are crucial for understanding disease spread, guiding treatment decisions, and prognosticating outcomes in breast cancer management [47].

The evaluation of tumor grade within the patient cohort reveals a distribution favoring Grade 2 tumors, indicating a predominance of moderately differentiated tumors, suggesting a moderate level of aggressiveness in most cases. The presence of well-differentiated and poorly differentiated tumors in smaller proportions reflects the biological diversity of breast cancer within this cohort. Grade 1 tumors are associated with a more favorable prognosis, while Grade 3 tumors, which are less differentiated, tend to exhibit more aggressive behavior and may require more intensive treatment [48].

The KI-67% variable, a marker for cellular proliferation, provides insight into the growth rate of tumors [49]. The analysis reveals a diverse range of values, indicative of varying tumor aggressiveness within the cohort. The variability in KI-67% depicts the heterogeneity in tumor proliferation rates among the patients. A higher KI-67% is generally indicative of a faster-growing, potentially more aggressive tumor, which could influence treatment decisions, particularly regarding the use of adjuvant chemotherapy in hormone receptor-positive, HER2-negative breast cancer [50].

The analysis of genetic markers, specifically BRCA mutations and other genetic mutations, provides crucial insights into the genetic predisposition and molecular characteristics of breast cancer in the study population. BRCA1 and BRCA2 genes are significant markers for increased breast cancer risk, with mutations in these genes associated with a higher likelihood of developing breast and ovarian cancers [51]. The proportion of patients not tested for BRCA and other genetic mutations suggests an opportunity for enhanced genetic screening and counseling in breast cancer management. For those tested, the identification of BRCA mutations in a subset of patients emphasizes the importance of genetic risk factors in breast cancer development [52]. Meanwhile, detecting other genetic mutations highlights the molecular diversity of breast cancer, which may have implications for personalized treatment strategies, including targeted therapies [53]. The findings advocate for the integration of genetic testing into the standard of care for breast cancer patients, facilitating risk stratification, informed decision-making regarding surveillance and preventive measures, and the optimization of therapeutic approaches based on genetic profiles. Furthermore, these results contribute to the growing body of knowledge regarding the genetic landscape of breast cancer, suggesting the need for ongoing research to uncover the full spectrum of genetic factors that contribute to breast cancer risk and progression [54].

Kaplan-Meier recurrence-free survival analysis was stratified by Oncotype DX risk groups, revealing no statistically significant difference in survival distributions across high, intermediate, and low-risk categories (p = 0.268231). The chi-square statistic of 2.631814, falling well below the critical value of 5.9915 for 95% confidence, further supports the absence of significant disparities in survival outcomes among these groups. Despite an observed effect size of 0.66 indicating some differentiation within the model (risk group stratification for predicting recurrence-free survival), the lack of statistical significance suggests that the Oncotype DX score, while useful for risk stratification, may not alone predict recurrence-free survival in this cohort. This finding prompts a deeper examination of additional factors that could influence prognosis and reflects the complexity of breast cancer recurrence risk assessment. Future research should consider incorporating more comprehensive models that include genetic, pathological, and clinical variables to predict better outcomes for patients stratified by Oncotype DX risk scores [55].

Limitations

The relatively small sample size of 95 patients may limit the generalizability of the study’s results to a broader population of breast cancer patients, particularly in a diverse setting like the UAE. Being a retrospective cohort study, it relies on historical medical records, which may introduce biases due to incomplete or inconsistent data recording. The retrospective nature also limits the ability to control for confounding variables prospectively. The study’s findings are based on patients from a single institution (Tawam Hospital), which may not reflect the diversity of breast cancer presentation and treatment outcomes across different healthcare facilities or regions within the UAE. The UAE’s unique demographic composition, with a significant expatriate population, introduces variability in genetic, cultural, and lifestyle factors that could influence breast cancer outcomes. This heterogeneity might affect the applicability of the Oncotype DX test’s predictive accuracy across different patient subgroups.

## Conclusions

This retrospective cohort study conducted at Tawam Hospital in the UAE offers valuable insights into the predictive accuracy of the Oncotype DX test for stratifying breast cancer patients by recurrence risk. While the test influenced treatment decisions, particularly regarding the use of chemotherapy, the analysis did not demonstrate a statistically significant correlation between Oncotype DX risk categories and actual recurrence events within the study’s cohort. These findings suggest that, although Oncotype DX is a powerful tool for personalizing breast cancer treatment, its predictive utility in the diverse and unique population of the UAE may require further evaluation. The study highlights the need for larger, more diverse cohort studies. It highlights the importance of considering regional and demographic differences when applying genomic testing to breast cancer management. Ultimately, enhancing the precision and applicability of genomic tests like Oncotype DX in diverse populations will be crucial for optimizing treatment strategies and improving global outcomes for breast cancer patients.
